# Drug users in Hanoi, Vietnam: factors associated with membership in community-based drug user groups

**DOI:** 10.1186/1477-7517-10-33

**Published:** 2013-11-22

**Authors:** Eleanor Hayes-Larson, Lauretta E Grau, Kaveh Khoshnood, Russell Barbour, Oanh Thi Hai Khuat, Robert Heimer

**Affiliations:** 1 Yale School of Public Health, 60 College Street, New Haven, CT 06511, USA; 2 Current address: Analysis Group, Inc, 10 Rockefeller Plaza, 15th Floor, New York, NY 10020, USA; 3 The Centre for Supporting Community Development Initiatives, B4 - 111, Tran Quang Dieu, O Cho Dua, Dong Da, Ha Noi, Vietnam

**Keywords:** Injection drug use, Harm reduction, Self-efficacy, Vietnam, Drug user groups, HIV, Hepatitis, Overdose, Quality of life

## Abstract

**Background:**

A syndemic conjoins injection drug use, incarceration, and HIV in Vietnam, where there is a need for programs that empower people who use drugs to minimize the harms thereby produced. Here we present a post-hoc evaluation of the organizing efforts of the Centre for Supporting Community Development Initiatives (SCDI) with two community-based drug user groups (CBGs) in Hanoi.

**Methods:**

Members (n = 188) of the CBGs were compared to non-member peers (n = 184) on demographic, psychosocial, behavioral and knowledge variables using a face-to-face structured interview that focused on issues of quality of life and harm reduction. Bivariate analyses were conducted, and variables significantly associated with membership at p < 0.10 were included in a multivariate model.

**Results:**

Variables associated with membership in the CBGs in the multivariate model included increased self-efficacy to get drug-related health care (OR 1.59, 1.24-2.04), increased quality of life in the psychological (OR 2.04, 1.07-3.93) and environmental (OR 2.54, 1.31-4.93) domains, and greater history of interactions with police about drugs (OR 3.15, 1.79-5.52). There was little difference between members and non-members on injection-related harms except in the domain of knowledge about opioid overdose. Among the 114 current injectors (30.6% of the sample), low rates of unsafe injection practices were reported, and low statistical power limited the ability to conclusively assess association with membership.

**Conclusions:**

Although the CBG members displayed higher levels of well-being and access to healthcare than non-members, further longitudinal study is required to determine if these are a result of membership. The CBGs should pay more attention towards meeting challenges in responding to specific health issues of those who continue to use drugs including HIV, hepatitis, and drug overdose.

## Introduction

In Vietnam, injection drug use and HIV are closely linked, and the HIV epidemic is concentrated in injection drug users (IDUs). Government documents report that 250,000 HIV cases have been reported through the end of 2011, there are an estimated 115,000-350,000 IDUs, and HIV prevalence among the IDUs is 13.4% nationwide. As many as 69% of incident cases occur in IDUs [1]. However, injection drug use has repercussions that extend well beyond the risk of HIV. Mortality rates among male IDUs are 13.4 times higher than the general male population after indirect standardization. Though the leading cause of death in IDUs was AIDS-related illness (31%), an additional 27% were caused by overdoses [1,2]. Beyond physical morbidity and mortality, drug use carries a significant mental health burden; an estimated 22.4% of opioid users in Vietnam have depression [3]. IDUs also report high levels of stigma from their communities, which are often amplified in the presence of HIV infection and make it difficult for IDUs to earn money, which in many cases places the burden of care on their families [4].

Vietnam is heavily influenced by Confucianism, which holds that the self should be considered last and that one should always devote one’s attention to the country and the family first. Communism, which was introduced into the country in the midst of harsh wars, echoed this norm in order to unify the country and mobilize people. People with addiction therefore have always been seen in a negative light as self-indulged, selfish, and spoiled. In addition, as evidence-based addiction treatment has only been introduced very recently with the initiation of methadone treatment programs (which so far have covered less than 10% of people with drug addiction), drug users need money to buy drugs, and they commit crimes. Although most of the crimes committed by drug users are petty crimes, they affect everyday life in Vietnam and drug users are thus perceived as dangerous, creating public chaos and deserving of punishment. Punitive measures therefore receive strong support from both policy makers and the general public [5]. Even for those drug users without HIV infection, the risks to their liberty are great through an extrajudicial administration system that can enforce mandatory incarceration for up to four years based solely on positive drug test results [6,7].

These interconnected ills demonstrate that IDUs in Vietnam are in need of comprehensive programs that can increase their quality of life, create more social support, and reduce their risks to health and safety. These programs, which include but extend beyond harm reduction, have not always been readily available. Indeed, the development of comprehensive programs designed to empower drug users is a relatively new phenomenon. It originated in drug user groups and a growing acceptance of a human rights framework that viewed criminalization of drug use as abuse [8-11]. This approach begins with the traditional individual-level definition of harm reduction and broadens it to include drug user empowerment as a development goal [12,13].

Historically, the government of Vietnam did not support harm reduction initiatives, and oversight of injection drug use policy fell to the Department of Social Evils Prevention in the Ministry of Labor, Invalids, and Social Affairs [14]. Drug users have often been sent to government detoxification centers, called 06 centers, where they could be incarcerated for up to four years at a time [7,15]. However, the official policy environment regarding injection drug use has shifted significantly in the last seven to ten years. In 2006, after seeing international evidence of the benefits of harm reduction programs, the Vietnamese government passed the Law on HIV/AIDS Prevention and Control, which officially sanctioned harm reduction programs including needle syringe programs (NSPs) and methadone treatment [16]. While many government officials, international partners, and community members say this revised policy approach is a clear improvement, they note that there is still conflict between drug control laws and HIV prevention laws [7,15,16]. Since harm reduction was written into the law in 2006, the government’s policy towards drug users has changed significantly, most notably through the decriminalization of drug use in the Amendment of Penal Code 2009 and the Administration Sanction Law 2012 following which a drug user can only be sent to a compulsory center by a duly constituted court. Currently, a Renovation Plan on Drug Treatment is being reviewed by the government, with approval possible as early as the beginning of 2014. However, in efforts to maintain “public order”, law enforcement tends to apply other existing legislation to keep drug users off the streets. While drug users are not supposed to be arrested for using drugs, they can still be arrested for possession of drugs or organizing drug use if more than one person is caught using at the same place. Until the Renovation Plan is approved, there are still more than 100 compulsory centers in the country and many drug users are still sent there.

High-level policy change does not always result in change on the ground. As many as 50,000 IDUs (25% of all IDUs) were in 06 centers in 2007, despite the fact that users who are sent to such centers have drug relapse rates of between 80 and 90% [15,17]. Scale-up of harm reduction programs has been slower than expected, coverage is still poor, and law enforcement and local communities still often do not understand the concept of harm reduction [16]. Although the government continues to officially oversee the response to the epidemic, community-based organizations are emerging as important players in expanding harm reduction programs and many are building on the empowerment model.

One of these emerging organizations is the Centre for Supporting Community Development Initiatives (SCDI), a Vietnamese NGO established in January 2010. SCDI aims to integrate expertise from a variety of fields including medicine, public health, psychology, law, economics, and education to implement and promote sustainable social development among marginalized and stigmatized populations in Vietnam. In collaboration with the Vietnam Civil Society Partnership Platform on AIDS, SCDI has supported the development of two community-based groups (CBGs) with storefronts for drug users in Hanoi.

These CBGs, called The Bullet Point (in the Hai Ba Trung District) and White Sand (in the Long Bien District), are largely organized and operated by current and former drug users. They were established in 2009 and currently receive most of their funding from SCDI although they raise some of their own funds through community service activities. The CBGs go beyond traditional harm reduction by offering a wide variety of services and activities including peer education on HIV, overdose prevention and safe sexual practices, providing support in detoxification, finding jobs, and accessing syringe exchange and medical care. The Bullet Point is closely linked with a group of sex workers and the sexual partners of drug users – forming a coalition named “Coming Home”. The White Sand is linked with Bright Future Network – a network of people living with HIV. The CBGs, which currently have over 500 members between them, also engage in advocacy in their communities to reduce stigma surrounding drug use and HIV. Other drug user CBGs and organizations are being developed around Vietnam based on the model these two CBGs provide.

There have been a small number of evaluations of peer-based programming for drug users in Vietnam, but more data about such programs are needed in order to demonstrate the impact of these programs and, if the results are promising, then serve as advocacy in expanding harm reduction and empowerment programs for drug users in Vietnam [3,14,15,18]. This report constitutes one of the first evaluations for such community-based programs in the city of Hanoi, and indeed in all of Vietnam. It adds to the body of knowledge about drug users in Hanoi by evaluating the impact of the CBGs supported by SCDI by interviewing members of the CBGs – both current and former drug users - and their peers who are not members. We focused on issues of general well-being, HIV, hepatitis, and overdose prevention knowledge, and interactions with the drug control system. We hypothesized that the CBGs would have positive effects on the lives of their members, and that for measures of quality of life, social support, and harm reduction-related knowledge, CBG members would surpass non-members. In addition, among the subset of members and non-members who were actively injecting at the time of the interview, we hypothesized that members would have lower levels of risky injection and greater access to sterile syringes.

## Methods

### Sampling

Subjects were recruited from January to December of 2011 in Hai Ba Trung and Long Bien, the two districts of Hanoi where the CBGs operate. After giving verbal informed consent, subjects completed a structured survey given by a trained interviewer from SCDI. The interview lasted approximately 50 minutes, and subjects were compensated 50,000 VND (about $2.50) for their participation.

This was not an intervention research study, and the cross sectional nature of the data collection required the use of a comparison group rather than baseline and follow-up data for members. Eligibility criteria included having ever used drugs illicitly, being at least eighteen years of age, and residing in a neighborhood served by one of the two CBGs. Members were defined as individuals who returned to the CBGs within three months of their first visit and appeared in the logs of the CBGs. The logbook recorded the participation of drug users in the program to detail attendance at CBG activities. When a drug user first participated, that individual was registered to the group and his/her information entered in the log. Because there are both hard copy and electronic records, a simple search of electronic records was employed to determine if individuals returned within three months of initial enrollment. This information was retrieved from the log and such individuals would be “qualified” as a member.

When members made contact with the center during the data collection period, they were offered the opportunity to participate in the study. Non-members were drug users from the same districts of Hanoi who had not had any contact with the CBGs or had only very recently made first contact and did not yet re-appear in the CBGs’ logs. They were accrued through community outreach by SCDI staff, referrals by members (for which members were compensated 30,000 VND, about $1.50), and referrals by other non-members who had participated in the study.

### Measures

Subjects were interviewed using a structured survey made up of existing, validated instruments. The survey assessed demographic information, psychosocial factors, knowledge regarding HIV, hepatitis, and overdose, exposures to the criminal justice system, and drug-using risk behaviors to get a full picture of the study population.

The World Health Organization Quality of Life-BREF (QoL) survey was used to assess self-reported quality of life, including overall quality of life, overall quality of health, and physical quality of life (as measured by self-reported pain, energy, sleep quality, mobility, activities, and medications), psychological (as measured by self-reported positive/negative emotions, self-esteem, body image, and spirituality), social (as measured by self-reported relationships, support, and sexual activity), and environmental (as measured by self-reported safety, home, finances, services, access to information, leisure, and transportation) domains. All items were measured using a 5-point Likert scale with higher scores indicating higher quality, and scoring of the survey was performed in accordance with the QoL manual [19].

Self-efficacy (self-confidence to perform a specific behavior/action) and social outcome expectancies (perceptions about others’ reaction to a specific behavior/action) were adapted from previously reported measures, and examined self-reported confidence in one’s abilities to get new syringes, refuse to participate in risky injection behaviors, access drug treatment and other medical care, and the perceptions of friends and family about drug use [20]. Questions assessing subjects’ knowledge regarding sexual transmission of HIV as well as the recognition and prevention of overdose and hepatitis infection were adapted from previously reported assessments and were scored as a percentage of questions answered correctly [21,22].

Questions about drug-using history and behaviors were adapted from earlier work in consultation with SCDI staff to ensure appropriateness to the Vietnamese drug-using population [23]. Subjects who reported injecting in the last thirty days were asked about their injection behaviors during that time, including frequency of injection, sharing drugs with others, reusing their own or another person’s syringes, passing used syringes to others, and injection hygiene.

The survey instrument was developed in English, translated to Vietnamese by a team of native Vietnamese speakers, and subsequently back-translated to ensure no loss of meaning in translation. It was administered to 53 subjects before a skip pattern was altered, one question was added, the definition of opioids was clarified, and then the remainder of the sample was collected.

### Data analysis

The outcome was defined as membership in the group, enabling us to examine multiple demographic, behavioral, and psychosocial factors that might differ between members and non-members of the CBGs. Demographic data included age, sex, education, and marital status, and where participants had slept in the last thirty days. Psychosocial factors were assessed as the mean score of the Likert scales. Knowledge was assessed as the percentage of true/false questions answered correctly out of four questions on sexual transmission of HIV, six questions on hepatitis, and eleven questions on overdose. Both incorrect and “Don’t know” responses were counted as wrong. Encounters with the criminal justice system were generally dichotomized as “ever” or “never”, but those who had been sent to a government detoxification center were asked how many times, and the mean number of times was reported. Those who had been to prison were asked if drugs were the cause of their imprisonment.

The analysis was performed in two stages. First, all variables were examined in bivariate analyses (t-tests for continuous variables and Chi-squared tests for categorical variables) for associations between the variable and member status. To minimize Type II error, all variables associated at the p < 0.10 level of significance were included in a multivariate logistic model with membership status as the dependent variable. The multivariate logistic regression was performed stepwise with manual backward selection, and only those variables that were significant at the p < 0.05 level of significance were included in the final model.

Because only a proportion of subjects had injected drugs in the last thirty days, a sub-analysis of those subjects was conducted, examining injection risk behaviors (past 30 days) and self-efficacy regarding getting new syringes and not engaging in risk behaviors. Risky behaviors were generally dichotomized as “never” or “at least once”. The number of injections per month, the proportion of those injections performed with new, unused needles, and the proportion of those injections performed alone were reported as means. Bivariate analyses (t-tests for continuous variables and Chi-squared tests for categorical variables) were performed to determine association between injection variables and membership.

Finally, because we were interested in the gaps in knowledge that exist in the population of drug users in Hai Ba Trung and Long Bien districts of Hanoi, the proportion of drug users answering each question correctly was assessed. The percentage of all subjects answering each knowledge question correctly was determined with a 95% confidence interval.

### Ethical review

Data were collected by SCDI for internal evaluation purposes, and therefore did not undergo review by an ethical review board. The analysis of de-identified data conducted in this paper was approved for exemption by the Human Investigations Committee of Yale University School of Medicine.

## Results

### Demographics and participant characteristics

The study recruited 372 current and former drug users from the Hai Ba Trung and Long Bien districts in Hanoi, Vietnam. Table 1 summarizes the major demographic characteristics of the study sample comparing members and non-members of the CBGs. The two groups did not differ significantly in age, age of first drug use, sex, education, marital status, or the type of lodging in the last thirty days. The mean age of subjects was 34.2 years, and the mean age of first drug use was 22.8 years. The majority of subjects were male (73.9%). Most participants (85.9%) had completed a middle or high school education. Of the remaining subjects, 8.1% had completed only elementary school, and 6.0% had attended any college. Exactly half of all subjects were married, another 20.4% were separated or divorced, few were widowed (3.2%), and the remainder had never married (26.3%). In the last thirty nights, nearly all subjects had slept in either their own home or the home of another (97.2%). The rest (2.8%) had slept in hospitals/clinics, government centers/prisons, or on the street.

**Table 1:** **Participant sociodemographic characteristics**^**a**^

**Variable**	**All 372 (100%)**	**Members 188 (50.5%)**	**Non-members 184 (49.5%)**	**P-value**
Age^b^	34.2 (6.9)	34.7 (6.7)	33.7 (7.2)	0.158
Age of first drug use^b^	22.8 (5.8)	22.3 (5.8)	23.2 (5.9)	0.110
Male	272 (73.9)	131 (70.8)	141 (77.0)	0.173
Education				0.515
Elementary	30 (8.1)	17 (9.1)	13 (7.1)	
Middle	146 (39.5)	69 (36.9)	77 (42.1)	
Some High school	66 (17.8)	39 (20.9)	27 (14.8)	
High school graduate	106 (28.6)	51 (27.3)	55 (30.0)	
Some College or more	22 (6.0)	11(5.9)	11 (6.0)	
Marital Status				0.130
Married	186 (50.0)	89 (47.3)	97 (52.7)	
Separated/divorced	76 (20.4)	46 (24.5)	30 (16.3)	
Widowed	12 (3.2)	8 (4.3)	4 (2.2)	
Never Married	98 (26.3)	45 (23.9)	53 (28.8)	
Sleep last 30 days^c^				0.536
Own or others’ home	353 (97.2)	181 (97.8)	172 (96.6)	
Other	10 (2.8)	4 (2.2)	6 (3.4)	

^a^Chi-squared test, given as N (column %) unless otherwise noted. Numbers and percentages may not sum to total due to missing data.

^b^t-test given as mean (standard deviation).

^c^Fisher’s Exact Test, given as N (column %).

As shown in Table 2, only two-thirds of the participants (68%) reported ever injecting drugs, and the number of injectors did not differ significantly by membership status. Fewer reported currently injecting (48.5%), and the proportion currently injecting was higher among non-members (55.3% vs. 42.2% of those ever injecting, p = 0.044). This is consistent with a significantly higher proportion of non-members also using opioids in any form during the past 30 days (57.1% vs. 44.1% of those ever injecting, p = 0.013).

**Table 2:** Participant drug use

**Variable**	**All 372 (100%)**	**Members 188 (50.5%)**	**Non-members 184 (49.5%)**	**P-value**
**Drug Use History**				
Age of first drug use^a^	22.8 (5.8)	22.3 (5.8)	23.2 (5.9)	0.110
Have ever injected drugs	236 (68.0)	118 (66.3)	118 (69.8)	0.481
**Current Drug Use**				
Used opioids in the last 30 days	187 (50.5)	82 (44.1)	105 (57.1)	**0.013**
Used amphetamines in the last 30 days	25 (6.9)	11 (5.9)	14 (7.9)	0.461
Injected in the last 30 days^b^	114 (48.5)	51 (42.2)	63 (55.3)	**0.044**

^a^t-test given as mean (standard deviation).

^b^Contingent on having ever injected drugs.

### Bivariate analyses

In the unadjusted bivariate analyses presented in Table 3, membership was associated with a statistically significant increase in all domains of quality of life (p < 0.001 to p = 0.007), self-efficacy to get healthcare (p < 0.001), and social outcome expectancies with family and friends (p = 0.002 and p < 0.001, respectively). Overall knowledge about HIV and hepatitis was low and did not differ significantly between members and non-members. On average, members answered 57.6% of HIV questions correctly, and non-members answered 56.2% correctly. Members answered 57.9% of hepatitis questions correctly, and non-members answered 54.2% correctly. Although members, on average, answered a larger percentage of the overdose knowledge questions correctly than did non-members (p = 0.007), knowledge of overdose was the lowest of all, with members answering a mean of only 37.6% of questions correctly and non-members answering only 33.2% correctly.

**Table 3:** **Associations between membership and psychosocial, knowledge, and criminal justice variables**^**a**^

**Variable**	**Bivariate analyses**			**Multivariate logistic model**		
	**Members 188 (50.5%)**	**Non-members 184 (49.5%)**	**P-value**	**aOR**	**95% CI**	**P-value**
Quality of Life (QOL)^b^						
Overall self-rated QOL	2.8 (0.7)	2.5 (0.7)	<0.001			
Overall self-rated health	3.0 (0.8)	2.6 (0.8)	<0.001			
QOL physical	3.1 (0.5)	3.0 (0.4)	0.007			
QOL psychological	3.2 (0.5)	2.8 (0.6)	<0.001	2.04	1.07-3.93	0.032
QOL social	3.1 (0.6)	2.8 (0.7)	<0.001			
QOL environmental	2.8 (0.6)	2.4 (0.5)	<0.001	2.54	1.31-4.93	0.006
Self Efficacy/Social Outcome Expectancy^c^						
Self-efficacy to get healthcare	2.7 (0.9)	2.1 (1.0)	<0.001	1.59	1.24-2.04	<0.001
Social Outcome Expectancy (family)	2.5 (1.2)	2.1 (1.1)	0.002			
Social Outcome Expectancy (friends)	2.6 (1.1)	2.2 (1.0)	<0.001			
Knowledge^d^						
HIV knowledge	57.6 (25.2)	56.2 (24.7)	0.607			
Hepatitis knowledge	57.9 (24.4)	54.2 (23.6)	0.145			
Overdose knowledge	37.6 (16.7)	33.2 (14.6)	0.007			
Drug Use, past 30 days						
Opioid Use	82 (44.1)	105 (57.1)	0.013			
Injection of Drugs	51 (27.1)	63 (34.2)	0.044			
Policing/Incarceration						
Ever Had Interactions with Police about Drugs^e^	147 (81.2)	123 (66.9)	0.002	3.15	1.79-5.52	<0.001
Ever in a Government Centre^e^	118 (65.2)	94 (52.8)	0.017			
No. Times in Government Centre (if ever)	1.4 (0.8)	1.5 (0.9)	0.912			
Ever sent to prison^e^	76 (41.8)	60 (32.6)	0.070			
Prison was about drug use (if ever in prison)^e^	61 (84.7)	46 (76.7)	0.239			

^a^t-test given as mean (standard deviation), unless otherwise noted. Numbers and percentages may not sum to total due to missing data.

^b^Responses for Likert scores 1–5 (Very poor, Poor, Neither poor nor good, Good, Very Good).

^c^Responses for Likert scores 1–5 (No confidence at all, Not very confident, Neither confident nor not confident, Somewhat confident, Totally confident).

^d^Percent of questions answered correctly (standard deviation).

^e^Chi-squared test given as N (column %).

Two of the five drug use items differed significantly between members and non-members. Both of these pertained to current use – use of an illicit opioid in any form in the past 30 days and injection of any drug in the past 30 days – and the frequency of these behaviors was lower among members. Neither of the drug use history items differed between members and non-members.

As shown in Table 3, a larger percentage of members than non-members (81.2% vs. 66.9%) had ever interacted with the police (i.e., arrests, questioning, urine testing, home visits) concerning drug-related activities (p = 0.002). A larger proportion of members (65.2%) than non-members (65.2% vs. 52.8%) had ever been in a government detoxification center (p = 0.017), but among those who had been sent to such a center, the average number of times sent did not differ between the two groups. Substantially more members than non-members (41.8% vs. 32.6%) had been in prison (p = 0.070), but a similar proportion of imprisonments were for reasons related to drugs.

### Multivariate model

In the multivariate model presented in Table 3, only four variables remained significantly associated with membership status. Reporting higher scores on psychological and environmental domains of the QoL was associated with membership in the CBGs (OR 2.04, 95% CI 1.07-3.93 and 2.54, 95% CI 1.31-4.93, respectively). Members also differed from non-members by their higher reported self-efficacy for getting drug-related healthcare (OR 1.59, 95% CI 1.24-2.04). Finally, self-reported police interaction involving drugs was the only criminal justice-related variable that remained significantly associated with membership (OR 3.15, 95% CI 1.79-5.52) in the multivariate model.

### Gaps in knowledge

Table 4 presents the knowledge items in the instrument and the percentages of respondents answering each question correctly. Given the lack of differences in knowledge scores, members and non-members were aggregated for this analysis. The proportion of subjects answering each HIV question correctly ranged from 45.2% (95% CI 40.1-50.3) to 66.9% (62.1-71.8). Hepatitis knowledge was slightly higher, ranging from 40.9% (35.8-46.0) to as high as 84.2% (80.4-87.9) answering each question correctly. Knowledge of overdose was the most varied, with the proportion of correct answers per question ranging from just 9.8% (6.7-12.8) to as high as 89.5% (86.4-92.6).

**Table 4:** Percentage of respondents answering knowledge questions correctly

***True/False item***	**Correct answer**	% correct*	***True/False item***	**Correct answer**	% correct*
HIV/AIDS KNOWLEDGE			OVERDOSE KNOWLEDGE		
When a couple decides that they are ONLY going to have sex with each other, they no longer need to use condoms to prevent HIV.	F	66.9 (62.1, 71.8)	Causing physical pain will help keep someone who is overdosing conscious or alive.	F	36.9 (31.9, 41.9)
The fewer sex partners you have in your life, the less likely you are to get HIV.	T	66.0 (61.2, 70.9)	Fatal ODs are more likely to happen when people use alone.	T	89.5 (86.4, 92.6)
People can still transmit the HIV/AIDS virus even if they test negative for the virus.	T	45.2 (40.1, 50.3)	People are more likely to have an OD if they use soon after getting out of a government center, prison, or detoxification program.	T	84.8 (81.1, 88.5)
Most people who have HIV know they have it.	F	52.1 (46.9, 57.2)	Most heroin OD deaths happen very quickly, in less than 15 minutes after taking the drugs.	F	9.8 (6.7, 12.8)
HEPATITIS KNOWLEDGE			It is easy to tell the difference between a “heavy nod” and a heroin overdose.	F	10.1 (7.0, 13.2)
Hepatitis can cause liver cancer.	T	72.1 (67.5, 76.7)	Sweating and anxiety are signs of a heroin OD.	F	58.0 (52.9, 63.1)
HIV is easier to transmit than hepatitis.	F	46.4 (41.3,51.6)	Injecting water can slow or reverse the effects of an opioid overdose	F	30.5 (25.7, 35.2)
You will be able to recognize if any individual is infected with hepatitis.	F	40.9 (35.8, 46.0)	Someone who is overdosing should be immediately placed on their back.	F	18.3 (14.3, 22.3)
Most people infected with hepatitis know that they have the disease.	F	46.4 (41.2, 51.6)	Naloxone is a medication for reversing the effects of a heroin OD.	T	47.7 (42.4, 53.0)
Drinking alcohol worsens the course of hepatitis C.	T	84.2 (80.4, 87.9)	Naloxone must be injected into a vein.	F	26.9 (22.2, 31.7)
You are more likely to get hepatitis B than hepatitis C by having unprotected sex.	T	54.3 (49.1, 49.4)	Naloxone will reverse a stimulant overdose.	F	24.8 (20.3, 29.4)

*Mean and 95% confidence interval.

### Sub-analysis of current injection drug users

Only 114 study participants had injected drugs within 30 days of the time of interview. The difference in percentages between members and non-members (27.1% and 34.2%, respectively) was not significant. Table 5 presents the bivariate analyses for variables regarding injection practices and injection-related self-efficacies. Members and non-members did not differ significantly for most of the behaviors assessed. However, without controlling for other variables, members were slightly older (p = 0.029), reported a larger proportion of injections with new needles (1.0 compared to 0.9, p = 0.036), and reported higher self-efficacy for getting new syringes (p = 0.004), refusing to share syringes (p = 0.006), and for not sharing water when injecting (p = 0.086). The sample size of active injectors was considered too small to conduct a multivariate analysis for the injection-related variables but suggests that additional research is needed to confirm and refine the findings from the small subsample of active injectors.

**Table 5:** **Associations between membership and psychosocial factors and injection risk behaviors in a subsample of IDUs only**^**a**^

**Variable**	**Members N = 51**	**Non-members N = 63**	**P-value**
**Age**	35.2 (6.5)	32.6 (6.1)	**0.029**
Age at first injection	24.9 (5.3)	24.3 (5.3)	0.560
Injections/month	49.0 (42.1)	49.4 (35.4)	0.948
Proportion of Injections with new needle	1.0 (0.1)	0.9 (0.2)	**0.036**
Proportion of Injections Alone	0.8 (0.3)	0.8 (0.3)	0.668
Split drugs^b^	19 (37.2)	31 (49.2)	0.201
Reused syringes^c,d^	3 (6.0)	7 (11.5)	0.507
Passed syringes to others^c,d^	1 (2.1)	4 (6.6)	0.382
Cleaned skin^d^			0.201
Never	28 (57.1)	42 (67.7)	
At least once	17 (34.7)	19 (30.6)	
Many times	4 (8.2)	1 (1.6)	
How Cleaned Skin^c^			0.101
Water	12 (52.2)	6 (31.6)	
Sponge/Alcohol Towel	4 (17.4)	4 (21.0)	
Anti-bacterial liquid	2 (8.7)	1 (5.3)	
Tongue/saliva	2 (8.7)	5 (26.3)	
Usually nothing	3 (13.0)	0 (0.0)	
Other	0 (0.0)	3 (15.8)	
How Stopped Bleeding^c^			0.127
Cotton/sponge	18 (39.1)	13 (22.4)	
Tissue	9 (19.6)	17 (29.3)	
Alcohol sponge	5 (10.9)	2 (3.4)	
Fingers	13 (28.3)	22 (37.9)	
Other	1 (2.2)	5 (6.9)	
Self-Efficacy^e^			
Get new syringes	3.8 (0.3)	3.4 (0.8)	**0.004**
Refuse to share syringes	3.5 (0.7)	3.1 (0.8)	**0.006**
Not share water	3.0 (1.1)	2.6 (1.2)	**0.086**

Bold p-values indicated variables that remained significant in the multivariate model.

^a^t-test given as mean (standard deviation) unless otherwise noted. Numbers and percentages may not sum to total due to missing data.

^b^Chi-squared test given as N (column %).

^c^Fisher’s Exact test given as N (column %).

^d^Dichotomized to Never/At Least Once, with At Least Once presented.

^e^Mean responses from Likert Score 1–5 (No confidence at all, Not very confident, Neither confident nor not confident, Somewhat confident, Totally confident).

## Discussion

The goal of this study was to determine the characteristics, psychosocial factors, and behaviors that differ between members of the two CBGs - The Bullet Point and White Sand - and drug users in the same districts of Hanoi who are not members and to test several hypotheses about the differences that membership in a CBG might produce. This report is focused on three hypotheses: (1) that CBG membership would have positive effects on the lives of their members, (2) that knowledge regarding syringe-transmitted infections (HIV and hepatitis) and drug overdose would be higher among members, and (3) that among active injectors, members would have lower levels within the 30 days prior to interview of risky injection practices and greater access to sterile syringes. The first and third hypotheses were confirmed while the second was not, although more data collection from active injectors (CBG members and non-members alike) is needed to strengthen our analysis of the effect of membership.

The demographic characteristics of members and non-members provide a picture of drug users in Hai Ba Trung and Long Bien that is generally consistent with earlier reports. The proportion of male drug user subjects (73.9%) is somewhat lower than in other studies, which have reported male/female ratios of 9:1 to 19:1 [5,24-26]. This may reflect one of three non-exclusive influences on participant recruitment: the reported increasing proportion of female drug users in Vietnam [5], an assumption of drug using to be a male phenomenon that limited other data collection efforts to recruit female drug users, or the heightened stigma experienced by female drug users that led them to hide their behaviors [27]. The lack of statistical differences in demographics between members and non-members provides evidence that members were similar to non-members prior to joining the CBGs. This in turn supports the hypothesis that differences observed in psychosocial and behavioral factors may be linked to participation in the activities of the CBGs although it was not possible to determine if these activities produced the difference.

The higher scores in the QoL psychological domain reported by members compared to non-members are important and encouraging. Several studies have shown a correlation between opioid substitution therapy and increased psychological QoL [28,29], and this study suggests that the CBGs under study may have a similar impact. In a country where only 1.3% of IDUs receive substitution therapy [17,30], CBGs may extend the reach of these mental health benefits to larger numbers drug users. Additionally, improved psychological QoL has been associated with reduced injection risk behaviors [31], which in turn affects transmission of HIV and hepatitis, suggesting that the benefits of the CBGs could spread beyond the psychological realm into physical health outcomes. However, we cannot rule out other explanations for the difference between members and non-members, including unknown factors such as presence of co-morbidities like depression and HIV infection, and employment status.

The reported higher self-efficacy among members in getting healthcare related to drug use (community based-treatment, substitution therapy, and medical care for problems related to drug use) has several possible explanations. First, because the CBGs offer referrals to healthcare, it is possible that the increased self-efficacy in members is due to already having successfully received a referral to drug-related healthcare [20]. Conversely, lower self-efficacy in non-members may result from their concerns about vulnerability to disclosure-related stigma from families and communities [28,32,33], perceptions of law enforcement activity [34], or disclosure of HIV status [35]. Membership in visible community groups such as the CBGs may mitigate these concerns. More research is needed to determine what other factors, both individual and environmental, are associated with increased self-efficacy to get healthcare. Because self-efficacy can be an important predictor of behavior [20,36,37], future interventions should aim to target those factors.

The association between membership and reporting interactions with the police can be explained by several non-exclusive factors. First, The Bullet Point and White Sand were able to conduct outreach at the 06 centers shortly after the CBGs opened, meaning that many of the members were recruited from a population where 100% of individuals had a history of interaction with the police. Second, drug users who have had an interaction with the police are more likely to be disclosed as drug users to friends and family as a result of this interaction. Conversely, undisclosed individuals risk revealing their drug user status to police and other community members by joining the CBGs; this may result in a reluctance to join among those who have not already had an interaction with police (Pham Thi Minh, personal communication, May 8, 2012). Finally, it is possible that because of more knowledge about the advocacy efforts of the CBGs, members felt more comfortable reporting interactions with the police to interviewers. The literature is conflicting as to whether police interference with community-based harm reduction efforts remains a problem [18,34]. More in-depth research is needed to understand the important role that law enforcement plays in the successes of and challenges faced by the CBGs under study.

Perhaps the most important finding was that the quality of life reported by subjects was also independently higher in members than in non-members for both the QoL environmental and psychological subscales. Rhodes and others have written about the “risk environment”, a construct encompassing factors exogenous to the individual, as a significant predictor of HIV prevalence and behavior change [38,39]. Our data indicate that the risk environment is lessened for members, which would suggest that the CBGs benefits might operate at a structural level. Although harm reduction programs often focus on individual factors like knowledge and changing individual behavior [40], their activities also work at the structural level to reduce negative consequences of drug abuse [22,41,42]. Advocacy efforts of the CBGs with law enforcement may allow members to feel more comfortable in their surroundings [43,44]. Members may also experience greater environmental security through factors not measured directly, including employment or HIV infection. Though some of these factors are likely affected by membership, greater environmental security may have made individuals more likely to join their CBG in the first place. Given the potential implications of improved environmental QoL on health outcomes, more research is needed on cause and effect in the relationship between membership and environmental QoL.

Both members and non-members had disappointingly low knowledge about HIV, hepatitis, and overdose. The reality may be worse than the raw scores indicated because the proportion of subjects answering a true/false question correctly usually overestimates the actual number who know the correct response – those knowing the answer provide it; those not knowing it may guess it [45]. Questions on HIV and hepatitis centered mainly on knowledge of sexual transmission due to concerns about generalized epidemics. While no clear trends emerged in HIV knowledge, no question was answered correctly by more than two-thirds of subjects. More subjects correctly answered questions about the course of virally-induced hepatic disease than questions about transmission and disease recognition. Given the already high rates of sexual transmission of HIV and hepatitis B in Vietnam, the low knowledge on sexual transmission is particularly concerning. More respondents were able to assess when an opioid overdose is likely to occur than were able to recognize its physical manifestations or how to manage one when it happens. While knowing when overdoses are likely to occur is important in preventing any overdose, poorly managed overdoses are a cause of unnecessary mortality and morbidity [46,47]. More overdose education efforts are needed to prevent and respond appropriately to them. More broadly, because behavior change is dependent upon knowledge, motivation, and necessary skills [48], improving knowledge about disease transmission through sex and overdose recognition and management should be priorities for the CBGs and all groups doing harm reduction in Hanoi and such efforts should be coupled with motivational and practical skills building training.

In the sub-analysis of IDUs, the frequencies of risk behaviors in both members and non-members were lower than in some recent reports [49,50]. While this could indicate a positive trend in injection practices, it could also be a result of reporting bias given the highly stigmatized nature of the behaviors under study or differences between our convenience sample and others previously accrued. Additionally, the small numbers of individuals reporting injection in the last thirty days, while a positive observation with respect to health outcomes, significantly reduced the power to determine differences between members and non-members. Despite statistically significant differences in the bivariate analyses in the proportion of injections performed with a new needle and a variety of risk reduction-related self-efficacy measures, the sample size was too small to conduct a multivariate analysis. Work with a larger sample size might provide more definitive evidence regarding the association between injection risk behaviors, self-efficacy, and membership in the CBGs under study.

This study is not without limitations. First and foremost, the cross-sectional nature of the study made it impossible to posit causal relationships between membership in the CBGs and differences in variables studied. Despite subjects being similar across all demographic variables examined, it is possible that additional and unknown factors or characteristics confounded or modified the associations examined. There is no way of knowing if the differences we observed between groups of members and non-members existed before members joined their CBG. Second, the sampling was not done at random, although several reports have indicated that snowball sampling can yield relatively representative samples of hidden populations [51]. However, our results may not be generalizable to drug-using populations outside of Hanoi. Third, many of the behaviors reported on are considered socially undesirable, and our reliance on self-report in face-to-face interviews may result in under-reporting of all behaviors, and of the riskiest behaviors in particular. Fourth, serological data confirming HIV and hepatitis B and C infection status were not collected. Although these data were not a direct interest in this study, it has been shown that HIV status can alter subject responses about self-efficacy and risk behaviors [29,35]. Additionally, it has been reported that injection risk behaviors may differ between HIV-infected and non-infected individuals in Vietnam, with HIV-positive individuals more willing to receive borrowed needles and share with other infected users [52]. Finally, there were challenges in measuring the degree of participation in the CBGs by those individuals we have characterized as members. Two CBGs were studied, and although they have largely identical structures, services, and programming, we were not able to analyze the CBGs separately to determine if characteristics and behaviors of members differed between the CBGs. Intensity of participation in the CBGs was also difficult to quantify but may provide important information in explaining the impact of the groups.

Looking forward, there are several possible avenues of research to pursue. A cohort study with longitudinal data would help to affirm a causal relationship between group membership and the variables studied here. Information on HIV and hepatitis B and C serologies and incidence could also be included, as slowing those epidemics is an important goal of harm reduction efforts. As previously mentioned, a larger sample size of active IDUs would give a clearer picture of the differences in risk behaviors between members and non-members. Finally, this study does not answer questions about stigma associated with drug use, and an evaluation of the effect of the advocacy efforts of the CBGs is needed. Such a study is in progress and will be reported once completed.

In conclusion, although there seem to be real and independent differences between members and non-members of The Bullet Point and White Sand groups across a variety of important psychosocial factors, the low knowledge regarding HIV, hepatitis, and overdose among non-members and members alike suggests room for improvement in the education components of the programming offered by the CBGs. Consistent with several theories of behavior change, the increased self-efficacy and quality of life (which seems to be present in members of the CBGs) can be combined with improved knowledge about risks and skills for negotiating those risks to provide services that might enhance the prevention of adverse outcomes like syringe-borne viral infections and overdose in drug users in Hanoi [35,38,48,53].

## Competing interests

The authors have no competing interests to report.

## Authors’ contributions

EHL conducted most of the analysis as part of her MPH thesis after the data had been collected. She received statistical assistance from Drs. KK and RB. Dr. LG contributed to the overall conception, design, and integration of the survey instrument and the creation of the study database. Dr. OTHK organized and led the data collection and assisted with translating and ensuring the comprehensibility of the survey instrument. She also secured the funding that allowed the establishment of The Bullet Point and White Sand CBGs. Dr. RH was responsible for the overall design and implementation of the survey and the analysis of the data. All authors contributed to the preparation of this manuscript with Ms. HL taking the lead and Dr. RH organizing the final draft. All authors read and approved the final manuscript.
